# Report on the Prevalent Diseases of the City of Chicago, from Nov. 1st, 1861, to February 1st, 1862, Including Two Cases of Puerperal Convulsions

**Published:** 1862-02

**Authors:** N. S. Davis

**Affiliations:** Member of the Sanitary Committee


### ARTICLE VI.

### REPORT ON THE PREVALENT DISEASES OF THE CITY OF CHICAGO,

FROM NOV. 22D, 1861, TO FEB. 1ST, 1862.,

INCLUDING TWO CASES OF PUERPURAL CONVULSIONS.

By N. S. DAVIS, M. D., Member of the Sanitary Com.

Read to the CHICAGO MEDICAL SOCIETY, Feb. 14th, 1862.

During the past two months we have noticed the prevalence of no disease meriting the name of epidemic; and the general sanitary condition of the city has been good.

Twice during the time, a sudden change from several days of severe cold to a temperature to make the roads and walks wet, with a high degree of moisture in the atmosphere, has been accompanied by a general prevalence of influenza. Most of the attacks were too slight to require medical attendance, but some were characterized by considerable fever, general muscular soreness, and sufficient inflammation of the mucous membrane of the respiratory passages to cause redness of the fauces, slight swelling of the tonsils and a harsh cough, producing soreness in the chest, and often a sore pain in the frontal region.

Most of these cases were promptly relieved by giving eight -

or ten grains of Dover's powder, with two of calomel, at bed time, and following it by a laxative the next morning—then using the following mixture for two or three days, viz:

℞—Chlorate of Potassa - - - - ʒij,

Tinct. of Belladonna - - - - ʒij,

Hydrochloric Acid - - - 30 gtts,

Simple syrup, Mint water, aa, ℥ij.

Mix, and take a teaspoonful every two or three hours.

In many cases of catarrhal sore-throat, which have occurred during the last month, by adding tincture of veratum viride or tincture of gelsemin to the last prescription, it was sufficient to remove all the unpleasant symptoms, without preceding its use by the Dover's powder and calomel.

I have seen but few cases of diptheria during the period under consideration, and those were of a mild character. The same may be said of scarlet fever and hooping-cough; but measles have been more prevalent, and in many instances, have been complicated with lobular pneumonia, and sometimes accompanied by a strongly marked typhoid condition of the system. The only fatal case that came under my observation, was in the person of a lady, over seventy years of age. In her case, at that period of the disease when the eruption was declining, she was attacked with severe dysenteric discharges, followed by well marked typhoid symptoms, gradual, but persistent engorgement of the lungs, and death.

During the last six weeks, I have met with several cases of pneumonia, both in children and adults; but they have presented no features worthy of special mention. Three of the adult cases were addicted to the excessive use of alcoholic beverages, and the disease was protracted in its duration and accompanied by muttering delirium, subsultus, and other typhoid symptoms. Three other cases were of that class which we sometimes style *congestive*. For instance, Mr. G——, on Fulton-street, was attacked on the 23d of January with rigors, followed by deep-seated pain in his right mammary region, accompanied by some febrile reaction, a sore pain through the forehead and temple, greatly aggravated by coughing, and considerable difficulty of breathing. I saw him for the first time on the following day, when I found his face deeply suffused with a venous-colored flush; the skin generally dry, but not much above the natural temperature; the pulse 110 per minute and soft; breathing short and oppressed; a sense of heaviness, with deep-seated soreness in the chest, greatly increased by coughing; expectoration scanty, but red from the intermixture of blood; the crepitant râle easily heard by auscultation, both in the mammary and axillary regions of the right side, and a moderately increased dulness on percussion. Believing the pneumonic disease to be still in the stage of congestion, I prescribed, with the hope of cutting short its further progress, as follows, viz:

℞—Pulv. Opium - - - - grs. x.,
Sulph Quinia - - - - grs. xx.,
Calomel - - - - - grs. xij.

Mix, and divide into six powders, of which one was given every three hours.

On the following day, (the 25th,) I found him sweating freely; face less flushed; pulse 90 per minute and soft; no pain in the chest, except on taking a full inspiration or coughing; cough less frequent and expectoration more copious, but only slightly tinged with blood. I directed powders, containing the same quantity of opium, with two grains of quinine, and one grain of pulverized bloodroot, instead of the calomel, to be given every four hours until six more had been taken.

On the 26th, finding the symptoms still further improved, I directed a laxative to move the bowels, to be followed by the following anodyne and expectorant mixture:

℞—Comp. Honey of Squills, Senega and Ant. ℥j,

Tincture of Bloodroot - - - - ℥ss,

Camph. Tinct. of Opium - - - - ℥jss.

Mix. Of which one tea-spoonful was to be given every four hours after the bowels had been evacuated.

I made him but two subsequent visits, the last on the 29th, when he was free from all symptoms of disease in the chest, and able to leave his bed.

I am satisfied that in malarious districts, and in patients who have suffered more or less from the effects of malaria, during the preceding summer and autumn, we meet with many cases of pneumonia, that may be promptly arrested by pretty full doses of quinine and opium, if used during the first 24 or 36 hours after the commencement of the attack. But after the stage of effusion or infiltration into the texture of the lung has supervened, no such speedy result can be obtained. And in the pneumonia of young children, opium must be given with more caution in every stage of the disease.

Cases of inflammatory rheumatism have been of frequent occurrence during the last two months. The treatment which has proved most promptly beneficial in my hands, has been as follows, viz:

℞—Tinct. Gelseminum - - - - ℥ss,<br>

Tinct. Verat. Viride - - - - ʒij.

Mix, and give from 12 to 15 drops every three hours; and

℞—Bicarb. Soda - - - - - - - ʒiij,<br>
Potassæ Acetas - - - - - - ʒiij.<br>
Water, - - - - - - - - - - ℥iv.

Mix, and give a dessert-spoonful between each of the doses of gelsemin and veratrum.

The idea of using the tinctures of gelseminum and veratrum combined, was suggested to me by Dr. Hoy, of Racine, Wisconsin, who claimed that a full sedative effect could be obtained by the mixture, with far less danger of producing vomiting than when veratrum is given alone.

During the period under consideration, I have met with two cases of Puerpural Convulsions—a form of disease so alarming and dangerous, that a brief account of the cases may not be unprofitable to the Society.

The first was Mrs. F., residing on North Wells-street; a short, fleshy, plethoric young women, in labor with her first child. She had been free from nausea or other debilitating symptoms during the period of utero-gestation; but during the last five or six weeks, there was some swelling of the face, and a moderate degree of œdema of the lower extremities. No tests had been applied to determine the quality of the urine. Her labor pains commenced on the morning of January 3d, but were not severe enough through the day to make much progress. I found her, in the evening, with regular pains about every five minutes; the os uteri moderately dilated and soft; the membranes slightly protruding; the presentation natural; and the labor apparently progressing with increasing force. The membranes soon after gave way, and and a large quantity of liquor amnii escaped. The head of the child, however, advanced slowly, and the patient exhibited considerable nervous restlessness, with frequent complaint of severe pain in the epigastrium. The latter, I was inclined to attribute to some indigestible food taken for supper, and gave a solution of soda, hoping it would relieve it. But as the labor advanced, it became aggravated, rather than otherwise.

By midnight, the os had receded, and the head of the child rested on the perineum, and although the pains continued regular and severe, the head advanced very little for two hours, and the patient became very restless, and complained still of the distress in the epigastrium. Thinking it unnecessary to protract her suffering, I requested the husband to go after my forceps; but he had scarcely left the house when she suddenly went into a general and severe convulsion. In a few minutes, the forceps arrived, and as the head was already distending the external organs, they were applied without difficulty, and the labor immediately completed. In a few minutes, she aroused from the stupor following the convulsion, and for three quarters of an hour appeared cheerful, and we began to feel confident that no further trouble would ensue. Suddenly, however, another severe convulsion occurred. I immediately corded the arm and took about 16 oz. of blood; and as soon as the stupor following the convulsions abated sufficiently to allow deglutition, I gave croton oil in doses of one drop every hour, applied cold to the head, and endeavored to keep her enough under the influence of chloroform, to prevent another convulsion. Despite these means, however, the convulsions continued to recur at intervals of from half an hour to an hour and a half, until four o'clock in the afternoon of the 4th, when they ceased, leaving the patient in a lethargic condition, breathing stertorously, with a pulse of 130 per minute, and soft, the face and tongue swollen, and the capillaries of the surface generally congested. Just before the convulsions ceased, the croton oil, of which six drops had been taken, aided by large enemas, produced two or three very copious evacuations from the bowels. These discharges were wholly unnoticed by the patient, and she did not recover from the lethargy until twenty-four hours later, on the afternoon of the 5th. From that time she improved rapidly, until the 9th, when she was seized with a chill, followed by all the symptoms of puerperal fever. To combat this, I gave a powder of two grains of pulverized opium and two of calomel every two hours, and four drops of tincture of veratrum viride between, until the pulse was reduced in frequency, the skin moist, and the patient sleeping. Then the interval between the doses was doubled in length, so as to merely maintain the effect already produced, until the abdominal pain and tenderness were entirely removed. The abdomen remained much distended and tympanitic several days, but she ultimately recovered, and both mother and child are now well.

Case 2d. Mrs. C———, about the end of the eighth month of pregnancy, was suddenly awakened about 1 o'clock, A. M., of the 7th of January, with extreme pain in the *epigastric* region.

The severity of the pain was such, that a physician was immediately summoned, who gave her some powders of sulphate of morphine, under the influence of which, she became quiet, and again slept for a few hours. On awaking from this sleep early in the morning, she was seized with a violent general convulsion. Dr. Ammerman was again called in, and made a vaginal examination. But finding no dilatation of the os uteri, or other sign of labor, he directed cold applications to the head, some cathartic pills to be aided by enemas, and waited for further developments. The convulsions continued to recur at intervals of from twenty minutes to an hour through the day. At first, she recovered her consciousness during the intervals between the paroxysms, but during all the latter part of the day, she was continually unconscious, and breathing more or less stertorously. About 5 o'clock, P. M., I was requested to see her in consultation with Dr. A. I found her as already described, unconscious, face swollen and livid; capillaries of the surface generally congested; extremities cool; pulse small, 120 per minute, weak; breathing hurried and noisy, from the mucous rattling in the trachea, and in a few minutes after entering the room, another general convulsion occurred. An examination, per vaginum, discovered no evidence of labor pains; the os uteri being open barely sufficient to admit the end of the finger; the anterior and posterior lips being still elongated, thick and unyielding; and the head of the child freely moveable above the brim of the pelvis. In attempting to dilate the os moderately with the finger, a small quantity of the liquor amnii was discharged. Although, perhaps of questionable propriety, after the convulsions had continued so long, I advised a bleeding from the arm; the administration of chloroform by inhalation; and the free application of extract of belladonna to the os and cervix of the uterus; the bowels having been already evacuated, and the bladder empty.

These measures being assented to by the attending physician; after some difficulty, owing to the thickness of the fatty tissue of the arm, I succeeded in opening a vein; but the blood was very dark color, and flowed from the vein so slowly, that not more than eight or ten ounces were obtained. We then introduced about 15 grs. of extract of belladonna mixed with ℥iij of simple cerate, into the vagina, carrying it as fully as possible up to the os and neck of the uterus; and endeavored to keep up a continuous, though moderate inhalation of chloroform. The convulsions continued to recur, though at gradually increasing intervals. About one hour after the introduction of the belladonna ointment, I thought I discerned in the occasional knitting of the eye-brows and certain motions of the body, evidences of slight uterine pains. On repeating the examination, per vaginum, the os uteri was found more open, and the anterior lip perfectly soft and yielding, while the posterior was still rigid. I introduced one or two drachms more of the belladonna ointment, and endeavored to apply it more directly to the posterior part of the os uteri. Slight uterine pains were now found to recur about every fifteen minutes. These were encouraged by gentle efforts to dilate the os with the finger, and we soon had the satisfaction of finding the labor actively progressing. About three hours after the first introduction of the belladonna into the vagina, the patient was delivered of a dead child, which appeared to be about 7½ or 8 months advanced in its development. The patient had one certainly, and perhaps two convulsions after the delivery, when they ceased to recur. She remained entirely unconscious, however, for nearly twenty-four hours longer. Soon after I met Dr. A. at the bedside of the patient, he introduced a catheter for the purpose of obtaining some urine to apply the tests for albumen, but the bladder being empty, none was obtained. Wishing to secure as early and efficient an action of the kidneys as possible, we directed six grains of iodide of potassa to be given in half a drachm each of fluid ext. conium and aqua camphora, every three hours, commencing as soon after delivery as the patient recovered the power of deglutition. She subsequently made a rapid, and perfect recovery. It seems to me that the local beneficial effects of the belladonna, in this case, were most strikingly manifested. I think it also lessened the severity and the frequency of the convulsions.

But in neither of the foregoing cases did the chloroform seem to produce any marked effect; and we doubt whether it is ever beneficial in that variety of puerperal convulsions accompanied by coma. There are cases met with, however, of a more hysterical character, in which there is little or no lethargy or unconsciousness between the convulsive paroxysms; and in such, I think the inhalation of chloroform may be of great service.

